# Book Reviews

**Published:** 1812-12

**Authors:** 


### CRITICAL ANALYSIS

### *OF RECENT PUBLICATIONS*

IN THE

### DIFFERENT BRANCHES OF PHYSIC, SURGERY, AND MEDICAL PHILOSOPHY.

*Treatise on the Influence of Climate on the Human Species; and on the Varieties of Man resulting from it; including an Account of the Criteria of Intelligence which the Form of the Head presents; and a Sketch of a rational System of Physiognomy as founded on Physiology. By* N. C. Pitna, M.D. &c. &c. Physician at Madeira. Longman and Co. 1812. 8vo. pp. 91. Plates.

THE subject of this treatise, though of acknowledged importance, has been much neglected by philosophers and naturalists. Medical practitioners especially, from their education and opportunities for observation, ought to be most intimately acquainted with human nature in all its varieties; yet little is accomplished towards the natural history of our species. We have still to range through series of isolated facts, and contradictory evidence; inconclusive deduction, and unsatisfactory hypothesis. It is still a question whether the whole human race be derived from one pair, and whether the varieties which exist in form, complexion, and feature, result from the influence of climate, or depend upon original conformation? The gradual operation of moral causes in counteracting the long-established consequences of physical agents, has not hitherto been sufficiently investigated. Some of those naturalists whose labors might have tended to elucidate the subject, have again obscured it by their fanciful conceptions, and began to generalize before they had attained a sufficient body of facts. Yet we think the knowledge of our species is advancing; the researches of Hunter,

Hunter, of Blumenbach, of Camper, of Cuvier, and of Gall, have thrown much light upon the natural history of man; on this subject the medical philosopher, whilst he smiles at some of their notions, may derive considerable instruction from the pages of Helvetius, of Buffon, of Lavater, of Hartley, of Kaimes, of Cabanis, and of Dr. Smith.

Our present author candidly avows that he is much indebted to this last writer, to Blumenbach, Camper, Le Brun, and to Mr. Walker, lecturer on anatomy and physiology at Edinburgh. From these sources indeed he has chiefly composed his work. But he has completed it in a neat manner, calculated to render the subject more familiar and popular than has heretofore been the case; and, though not strictly medical, we deem it well worth the attention of our readers.

The first part treats of the Influence of Climate in general, and, from its marked and obvious effects, the author has no difficulty in assigning it as a sufficient cause for the varieties in the human species.

"Every permanent and characteristic variety in human nature, (he observes,) is effected by slow and almost imperceptible gradations. Great and sudden changes seem too violent for the delicate constitution of man; and, in reality, instead of merely altering, they always tend to destroy, the system. But changes, the effects of which blend with the general actions of the body, and ultimately form the character of a climate or a nation, are progressively carried on through several generations, till the causes that produce them have attained their utmost operation, by becoming perfectly congenial to the system. In this way, the minutest causes, acting constantly, and during a long period of time, will necessarily create great and conspicuous differences among mankind.

"In tracing the globe from the pole to the equator, we observe a gradation in the complexion of man, nearly in proportion to the latitude of the country he inhabits. Immediately below the arctic circle, a high and sanguine color prevails. From this, we descend to the red, blended with white; and thence, towards the line, succeed the brown, the olive, the tawny, and at length the black.

"These gradations are sometimes more and sometimes less sudden. The women of the province of Biscay, for instance, are very fair: those of Grenada, on the contrary, subfuscous, 'so that,' says Ol. Toree, 'in this more southern province, the pictures of the Virgin are painted of the same provincial color.'

"The same distance from the sun, however, does not, in every region indicate the same temperature of climate. Some secondary causes, correcting and limiting its influence, must be taken into consideration. The elevation of the land, its vicinity to the sea, or to great lakes and rivers, the nature of the soil, the state of cultivation, the course of periodical winds, and many other circumstances, enter into this view."

From these general observations, the author concludes,

"That there is a general ratio of heat and cold, which chiefly forms what we call—climate, and a consequent general resemblance of nations, according to the latitude from the equator, subject, however, to innumerable varieties from the infinite combinations of the circumstances suggested."

He supports his opinion by various facts, such as, that summer darkens the skin of man, and the cold of winter excites a sanguine color; the Æthiop is white at birth, and becomes not black till exposed to the light: when heat or cold predominates in any region, it impresses, in the same proportion, a permanent and characteristic color, &c. &c.

"No individual example of the influence of climate can carry with it greater force than that of the Jews. Descended from one stock, prohibited by their most sacred institutions from intermarrying with other nations, and yet dispersed into every country on the globe, this one people is marked with the colors of all:—fair in Britain and Germany, brown in France and in Turkey, swarthy in Portugal and in Spain, olive in Syria and Chaldea, tawny or copper-colored in Arabia and in Egypt."

The inhabitants of the United States of America are cited as still more strongly exemplifying the influence of climate.

"A certain countenance of paleness and of softness strikes a traveller from Britain, the moment he arrives on that shore. A degree of sallowness is visible to him; which, through familiarity, or the want of a general standard of complexion, hardly attracts the observation of the Americans themselves. The effect is more obvious in the southern than in the northern states; and more in the lower and more laboring classes of people, than in families of easy fortune, who possess the means and the inclination to protect their complexion."

This subject, undoubtedly, is curious, and deserves more consideration than we can devote to it; but, although all the facts which our author has brought to bear upon it are directly favorable to his theory, which indeed was long ago embraced by ourselves, probably from investigating the same sources of information, and also from large evidence gleaned from the accounts of numerous travellers; we have lately been less confident in our opinion. M. Humboldt, in his political essay upon New Spain, has advanced some very staggering facts, which we wish Dr. Pitna had explained, for they are diametrically opposed to his theory. We have had no opportunity of seeing the original work of M. Humboldt, but have no hesitation in transcribing his opinion from the Edinburgh Review, as we cannot suspect any misrepresentation from that highly respectable quarter. The scientific traveller remarks, that climate which has such influence fluence on the European race, has little or no effect on the complexion of the Indians. Some tribes are darker than others; but the difference is quite independent of climate. Those who live on the Rio Negro are darker than those who inhabit the banks of the Lower Orinoco, though they enjoy a much cooler temperature. Near the sources of the Orinoco, there are tribes of a very light complexion, surrounded by others of a much more swarthy color. The Indians who live in Chili, and on the tops of the Andes, are as dark as the inhabitants of the plains, though the former are clothed, and the latter go almost naked; and those parts of their body which are constantly covered are not lighter than those which are continually exposed to the sun and air. The Mexican Indians, though they inhabit the same climate with the natives of Quito, are of a darker color; and those who live as far north as the river Gila, are swarthier than the inhabitants of Goatemala. M. Humboldt has also observed, that, in Mexico, Peru, Quito, Caracas, and other provinces of Spanish America, the children of the Indians are copper-colored from the moment of their birth; and the Caciques, who are constantly clothed, have all parts of their body of the same copper-color, except the palms of their hands and the soles of their feet. It appears, therefore, that the copper-color of the aborigines of America is independent of climate; and the same is probably true with respect to the darker complexion of the Negroes and Hindoos.

These facts, stated by such an intelligent observer as M. Humboldt, cannot easily be got over, we are obliged to admit them, and, however reluctantly, relinquish our former hypothesis, or at least suspend our decision. It is to be regretted that Dr. Pitna has followed the old beaten track on this subject, without advancing any new object for discussion, and even altogether neglecting those facts which we have just quoted on the authority of M. Humboldt, and which have long obtained a most extensive circulation through the medium of the Edinburgh Review. The learned author, indeed, backs his argument with appealing to an authority which we dare not call in question; he says, it is unphilosophical in the extreme to have recourse, in explaining the varieties of man, to the hypothesis of several original species, because it opposes a tenet of religion. We are not at present disposed to consider the connection between religion and philosophy, but we assert the privilege and the practice of a philosopher, which is to strip his facts naked, divest them of their adventitious coating, whether of filth or of finery, and from them fairly deduce his theory, which cannot be true if one fact directly contradict another. Thus, the the author, with probably a very large majority on his side, asserts all mankind originally sprung from one pair, and attributes the varieties in form and color of the human species, whether in the European, the African, or the American, to the influence of climate. But, unfortunately for this hypothesis, an intelligent traveller and acute observer, finds whole nations of people who appear to be totally uninfluenced by climate. Now, if we admit this to be true, what becomes of the hypothesis? The author's scriptural allusion may also be met with a query; if mankind consisted of one family, why was Cain, when obliged to wander as a vagabond on the face of the earth, afraid of meeting with strange people who might destroy him?

In the second part of his work, Dr. Pitna considers the particular effects which climate produces, and the classification of the species which result from it; and presents us with the opinions of Camper and Blumenbach, without any addition of his own that we can discover.

The third part contains some ingenious observations upon "the formation of the head, and the criteria of intellect which it presents."

The author, we suppose from the doctrine advanced by Mr. Walker, lays down some general principles of physiognomy, founded on physiology, which deserve examination.

"Thus," he contends that, "organs of sense greatly developed, in comparison to the cerebral and cerebellic cavities, indicate the pre-eminence of sensation, and a diminished degree of intellect and voluntary power; the cerebral cavity greatly developed, in comparison to the organs of sense and the cerebellic cavity, indicates the pre-eminence of intellect, and a diminished degree of sensation and voluntary power; and the cerebellic cavity greatly developed, in comparison to the organs of sense and cerebral cavity, indicates the pre-eminence of voluntary power, and a diminished degree of sensation and intellect.

"Thus, we possess the means of ascertaining the degrees of the three simple powers, sensation, mental operation, and volition, in man, and all the superior animals, in whatever proportion they may be combined.

"Moreover, wherever these organs are elevated, there their functions are brilliant or intense; wherever they are wide, there they are stable and permanent. Thus, the elevated cerebrum indicates genius and imagination; the broad one, the more mathematical talent: when the cerebrum is longest anteriorly, then perception and observation excel; and when it is longest posteriorly, then reflection and passion excel: when the cerebrum is elevated before and depressed behind, then the perceptions are brilliant or intense, the reflections less so; and when it is depressed before and elevated behind, then the perceptions are less, the reflections more intense; when when the cerebrum is broad before and narrow behind, then the perceptions are more permanent, the reflections less so; and when it is narrow before and broad behind, then the perceptions are less permanent, the reflections more so."

Towards the conclusion of this section, we think our author, who is chiefly indebted for his performance to the scientific labors of others, is rather too severe upon Dr. Gall; whatever absurdities his system may contain, he must be regarded as an original observer and experimenter, and some very intelligent men even are beginning to find more truth in his opinions, than at first they were willing to allow. But Dr. Pitna attributes any approximation made by the ingenious physiologist towards truth entirely to accident or empiricism; and hardly ever fails, in naming him or his system, to apply the epithets absurd, empirical, &c.

The fourth part, for which the author also acknowledges his obligations to Mr. Walker, contains some curious physiognomical details. We have not space sufficient to permit us to extract many of these, but the article on noses ought not to be passed over, as it restores the organ to its proper dignity, which it has not enjoyed since the fatal decree of Lavater—that it is not an important feature in physiognomical expression.

"*The magnitude of the nose,* when the organ is, at the same time, well constructed, indicates the greater capability of the sense of smell. A nose which is very broad, and at the same time moderately elevated, is evidently an organ by means of which the most extensive, and consequently the strongest, impressions are received. Hence, those possessing such a form of organ, are generally of firm and strenuous character. This is not to be wondered at, since man is the very creature of the impressions which he receives, and since all his most complex mental operations must depend upon the strength, weakness, &c. of his individual sensations.

"*A nose which is very flat,* is obviously rather adapted for heavier odors, which by its means will pass rather to the inferior than to the superior convoluted bone within the nasal cavity, and which forms the proper organ of smell, as well as the superior. Hence, as mental operation is throughout dependent upon sensation, the persons having such a form of organ, are generally of a character correspondingly depressed, or rather may be termed manageable as to character.

"*A nose which is very elevated,* or of that form called Roman, is evidently better adapted for the lighter and acuter odors, which will pass to the upper part of the organ of smell. Hence, such persons are almost always more haughty and commanding in character.

"*A nose which is long* will more slowly transmit odors, which will, therefore, make a less powerful impression upon the proper organ organ of smell. Hence, persons with long noses are always slower, and milder in their character.

"*A nose which is short*, and more especially turned up at the extremity, will more rapidly and acutely transmit impressions. Hence, persons with such a nose are always quick, pert, and even impudent. The same is remarkable among quadrupeds: the pug dog, for instance, has such a form of nose, and precisely such moral habits. This form of nose is particularly unseemly, from its exposing those cavities of the organ which modesty would conceal. Hence, persons possessing it, are not merely impudent, but often indelicate and filthy."

After this tirade, can the author modestly call Dr. Gall absurd?

The fifth and concluding part consists of five pages, and this Dr. Pitna claims as being peculiarly his own. It professes to treat of the passions in particular; we see nothing extraordinary in it, being merely a brief description of the appearance of the countenance during the operation of certain passions or emotions. Upon the whole, we consider this an ingenious essay, and would advise the author to regard it as such, and proceed in his career; the performance evidently is young, but we think it affords promise of further growth, in which we wish it may succeed, and gather strength as well as bulk.

---

*Edinburgh Medical and Surgical Journal, No.* XXXI.

I. *Observations on the Opinions of Mr. Wilson and Dr. Adams, respecting the existence of the Venereal Disease in the Island of Otaheite; by* H. Edmonstone, Surgeon.—The origin of Syphilis, and the question of its existence in Europe long prior to the discovery of America, has excited much learned discussion; and the fact is still left undetermined. Well may the appearance of this disease, first in the obscurity of the latter end of the fifteenth century, be a matter of doubt, if we cannot now determine whether it exists or no in an island with which Europeans are tolerably well acquainted; and where the manners and morals of the inhabitants are most favorable to its propagation, if it does exist. Mr. Wilson, surgeon of the ship Porpoise, asserts as a fact, in 1806, that the venereal disease did not exist in Otaheite; and Dr. Adams, in his commentary on Mr. Hunter's Treatise, argues for the same conclusion. Mr. Edmonstone supports the opposite proposition with force and ingenuity, and offers arguments to prove that the disease might have been exterminated in Otaheite prior to Mr. Wilson's visit in 1806.

"Mr. Wilson lays much stress on the improbability of the venereal disease being eradicated, after having once been so common, 'even,'

'even,' says he, 'if the inhabitants were in possession of a specific remedy, of which I am well assured they are not.'

"That they were in possession of no antidote of powers equal to those of mercury, is certain, but that they were destitute of every means capable of exerting a partial influence over the disorder, is not so apparent. Waving, however, all contest on this point, and admitting that the natives of Otaheite never knew of any specific for lues venerea, the extermination of the disease would not seem, on that account alone, to be impracticable. The terror which its first introduction excited in the inhabitants, and the system of seclusion and abandonment to which they had immediate recourse, 'lest,' as Captain Cook observes, 'the calamity should spread by contagion,' are circumstances that render it by no means difficult to comprehend the possibility of setting limits to its propagation, if not of extinguishing the malady altogether.

"A plan of this kind, if duly acted upon in such a situation as Otaheite, an island of very limited extent, and where concealment was not easily practised, would, in a short time, be productive of the most beneficial consequences in this respect. All voyagers bear witness to the unlimited power of the chiefs in the South Sea islands, and especially to the rigid manner in which the *taboo* is enforced, and the impossibility of infringing its laws. This, which is the most perfect system of quarantine or embargo, of which, perhaps, there is any instance, is made to operate with equal celerity on any number of people, and to any distance, from that of a few yards to a whole district or island.

"It may even be urged, with some degree of confidence, that the very absence of a remedy, and the consequently hopeless nature of the disease, would form the strongest reason for compelling the infected to withdraw from the healthy, and for inducing the latter to abstain from every species of intercourse by which the disease might be imparted. Nay, it is probably not hazarding too much to allege, that were circumstances of a similar description to occur in European countries, where detection is so much more difficult, the extirpation of lues venerea would infallibly take place, though a considerable period must necessarily elapse before it could be effected. It is the assurance amongst us of possessing the means of cure, that takes away much of the dread of exposure to infection. Let it once be understood that the disease is irremediable, and the causes which tend to perpetuate its ravages would soon cease to operate. It should be kept in view, also, that in these distant islands there did not exist, except during the visits of Europeans, the incitements to sexual intercourse on the part of the diseased, which, in more civilized regions, arise from motives of interest or imperious necessity, and which have so direct and powerful a tendency to keep alive the infection. It appears, from the account of Lieutenant Watts, which appears in the history of governor Philips's voyage to New South Wales in 1788, that the island of Otaheite had not been visited by any European for nearly ten years, a period fully capable of occasioning a very considerable decrease in the number of the diseased; the principal source of infection being entirely cut off, and supposing the precautionary measures to have been duly persevered in. It is even possible to trace the happy effects which the system of internal quarantine had produced in the short space of time that intervened between the introduction of the disease by Bougainville's people, and the arrival of the Endeavour, and still more remarkably during the interval between Cook's first and second voyage.

"As a farther proof, if, indeed, any were wanting, of what such a practice can accomplish, it is only necessary to advert to what Mr. Wilson himself has written. Upon no other principle can the unfrequency of gonorrhœa, during the first visit of the Porpoise, be explained; for, without some regulation of this kind having been extended even to that comparatively trifling complaint, there is no adequate cause to account for the rareness of the disease, where the intercourse of the sexes was unlimited. On the contrary, the disease, under such circumstances, ought to have increased. We find, also, that when a fresh importation of infectious matter took place, or when the temptations to promiscuous venery were present in greater force, the disorder immediately spread itself.

"The fact, therefore, of the non-existence of the disease in the island of Otaheite, even if it were incontrovertibly established, is far from amounting to a demonstration that the disease had not existed there at some former period."

II. *Case of Urinary Calculus; by* Alexander Jack, Surgeon.—This case of calculus, which had existed many years without occasioning the usual symptoms, became suddenly painful, and terminated fatally in a few months. On dissection it appeared probable that the stone had been retained at the fundus of the bladder; but, by some accident, or, as here stated, by some strong spasmodic action of the bladder, it had been separated from its attachment, or from the cyst in which it was contained; and then the severe symptoms of calculus appeared. We would draw one practical inference from this fact. Those who are suspected or known to have calculus, though in a quiescent state, should avoid severe exertion, or any exercise that may occasion the stone to be loosened from the part to which it is attached by a partial inclosure in a cyst, or by any other mode.

III. *An Account of a Case of Lythotomy, with practical Remarks; by* James Barlow, Surgeon.—There are three reasons which induce us to insert the greater part of Mr. Barlow's paper—the precision of the detail, the practical facts, and the study it affords for the young surgeon.

A corpulent robust man, sixty years of age, had, for some years, discharged mucus and blood by the urethra, and was suspected to have stone in the bladder. In November 1811, Mr. Barlow was enabled to ascertain the fact; and here we take up his narrative.

"On

"On my arrival, I was informed that he had not been able to evacuate his urine for nearly two preceding days and nights. On laying my hand on the abdomen, the patient complained of a considerable degree of pain in the region of the bladder, which was connected with tension, and the scrotum and ambient parts were of a dark livid color. The catheter was immediately introduced, and my former opinion fully confirmed by the instrument striking against a stone, and was also notified by the sound emitted to the ear very distinctly. Nearly two quarts of dark-colored urine were drawn off, which afforded temporary relief; the warm bath was recommended, a laxative glyster was administered, and an aperient mixture directed to be taken in divided doses, which produced several copious evacuations by stool, and reduced the tension, and considerably relieved the soreness of the abdominal region. Nevertheless, the retention of urine still continued, caused, I apprehend, by a calculus lodged in the vicinity of the neck of the bladder; for every time the catheter was passed, it was resisted by the presence of a stone, and little or no water could be extracted without first pushing the point of the instrument against it, and raising the stone from its situation, and keeping the instrument in this position till the bladder was emptied. By this manœuvre the impediment was surmounted, and the urine evacuated once or twice every twelve hours during several succeeding days, by the use of the common silver catheter, until I prevailed on my patient to be removed to the town (Blackburn), where I had an opportunity of paying more particular attention to the urgency of the case. On his arrival, I introduced a flexible metallic catheter into the bladder, fitted with a small cork to plug up the end; the fore-finger being passed up the rectum, served to bend the apex of the instrument under the arch of the pubes, where it was permanently fixed, so that he could evacuate his urine *ad libitum.*

"The indispensableness of the finger in the rectum, whilst passing the instrument, afforded me an opportunity of ascertaining the morbid indurated state of the prostate gland, which was greatly enlarged, and in a very rigid condition.*

"Notwithstanding

---

* The symptoms of stone in the bladder, and a morbid condition of the prostate gland, being in some respects similar, it is highly necessary that the surgeon should invariably pass the finger up the rectum prior to the operation, and, if the gland be enlarged, it will be discovered with the greatest facility. By such timely investigation, the surgeon will be governed in the various stages of the operation, and thereby be enabled to form a pretty accurate idea of the determinate extent of the incision of the neck of the bladder, and necessary extension of the blade of the bistouri, prior to its introduction into the bladder.

The presence of a stone in the bladder may, generally, by attention, be distinguished from an irritable state of that viscus; for in the

"Notwithstanding the antiphlogistic regimen was rigorously adhered to, there remained a considerable degree of soreness on the region of the pubes, attended with quick pulse and fever, insomuch that I did not then propose the operation of lithotomy, being aware of the consequences that might ensue from the attendant symptoms, excited by consequent irritation, the frequent effects of calculi, and a long distended state of the bladder; nevertheless, these unfavorable symptoms gradually abated, together with the tension of the abdomen, and the operation became admissible;* and, after being determined on, and the preparatory regimen adopted, I performed it on the 17th instant (November), in the presence of two assisting surgeons and the necessary attendants. The patient being placed and secured in a horizontal position upon a study table of commodious height, and supported by pillows with the breech projecting over the edge of the table, the first stages of the operation were conducted in the usual manner, and with tolerable facility. On the membranous portion of the urethra being laid open with the scalpel to the commencement of the prostate gland, the beak of the bistouri caché was inserted into the groove of the staff, the handle of the staff was taken hold of with the left hand, and raised from the right groin of the patient to nearly a right angle with the body; the bistouri was then carried gently forwards into the bladder, and the staff taken out; the cutting edge of the bistouri being turned laterally towards the left ischia of the patient, and raised from its sheath, it was withdrawn† nearly in a horizontal direction; and, in executing this

---

the former affection, and during the discharge of urine, the pain increases till the last drop is voided; whereas, in the latter case, relief is observed in a reverse ratio,—for, as soon as the water begins to flow, the patient is somewhat relieved, and when the whole is evacuated there is no pain left, and the functions of the bladder are usually restored.

* Daily observation evinces, that simple irritation excited in the bladder, by the presence of a stone in that viscus, not unfrequently subsides on the primary exciting cause being removed by its extraction, without impeding its functions.

It does not appear that a low degree, even of frequent irritation, excited by a stone in the bladder, is altogether unfavorable to the success of the operation of lithotomy, when performed during an interval of cessation from pain; but, if there exist any nephritic affection, the danger is then manifestly increased, and the operation should be undertaken with great caution, and deferred, if possible, till such pain wholly ceases, and the consequent irritation of the system subsides.

† The bistouri caché possesses an important advantage over the gorget, which no construction of that instrument can possibly surmount; for the surgeon has it in his power to adapt the blade of the bistouri to the exigency of every individual case, prior to its introduction this step of the operation, I perceived an unusual resistance* and grating sensation, as if cutting through a cartilaginous substance. The fore-finger of the left hand was now passed as high as possible into the bladder, through the opening made by the bistouri, and with difficulty the surface of a stone was felt; for, owing to the man's state of corpulency, the greatest part of the hand became buried in the wound. The forceps were then carefully introduced by the side of the finger, which served as a guide to detect the stone. The finger being withdrawn, the stone was seized by the blades; but from the great expansion of the handles, I was led to believe that the calculus was either very large, or otherwise taken hold of in an unfavorable direction. To ascertain this incident, I endeavored to reach the stone by insinuating the finger betwixt the extended blades of the forceps, but was opposed by the bulk of the prostate gland; for it appeared to occupy so considerable a space, that its extent could not be wholly traced by the finger in any direction. I therefore judged it expedient to let go the stone, and attempt to seize it in a less diameter, and, after using every possible means in my power, I was obliged to abandon this project; and the extent and rigidity of the prostate, and its unyielding condition, induced me to enlarge the incision; for on every attempt to extract the stone, the body of the gland was brought forwards into sight, and appeared to completely wedge up the space betwixt the two rami ischii. Thus situated, and whilst the left hand was employed in gently drawing forwards the forceps along with the stone, the right was engaged in dilating the wound with the scalpel in a line with the external incision, where the resistance opposed the chief obstacle: in this manner sufficient room was made, and the transmission of the stone effected. It was of an oval shape, and its long diameter 2·25, and its short 1·75 inches. A female sound was immediately passed into the bladder, and another stone detected larger than the first, and which was extracted with proportionate difficulty. It was also oval, but measured 2·6 inches one way, and 2·1 the other. From the different situations in which I had an opportunity of recognising the prostate gland of this patient, both by the finger passed up the rectum and through the wound in perineo, its lateral lobes evidently projected considerably on the rectum, and it appeared the shape and size of the gizzard of a goose.† Several arteries were divided in the

---

introduction into the bladder, and, should the stone be suspected to be of unusual magnitude, or less than what was at first imagined, the screw of the handle may be moved whilst the apex is in the bladder, and the cutting part regulated accordingly.

* The enlargement of the prostate gland, in this instance, seems most probably to have been occasioned by the irritation which the long retention of the stones in the bladder had excited on the functions of that organ.

† Although a morbid enlargement of this gland may induce an unfavorable the operation, which required the ligature; and there was a considerable oozing of blood, which appeared to come from the divided edges of the prostate gland. A canula was introduced into the wound, which, by its pressure on the incised portions of the gland, prevented the blood from making its way into the bladder, and soon stopped the bleeding.

"A plaster of lint, spread with cerate, was applied to the wound; the patient was then conveyed to bed, and his knees brought together and secured by means of a tape passed round his thighs. A draught, composed of sixty drops of tinct. opii was administered, and the patient left to take repose. On calling in the evening, I was informed that the medicine had not produced sleep: he appeared restless, with quick pulse. There was no tension or pain about the region of the bladder, nor any hæmorrhage from the wound, and the urine flowed *guttatim* through the canula without interruption.

"A warm bath was immediately procured in the room, into which he was put, and remained twenty-five minutes, which afforded some temporary relief, but without producing syncope, or diminishing the vibratory force of arterial action. On being removed to bed, the opiate draught was repeated, but did not induce the least inclination to sleep the whole of the night. In the morning, the canula was removed, and the wound dressed as before. A saline mixture, with antimonial wine, was directed to be taken. An aperient glyster was administered for several succeeding days, and occasional purgatives exhibited to stimulate the torpid disposition of the intestines, all which produced their desired effects. The warm bath was repeated twice every twenty-four hours for ten days successively, and the antiphlogistic plan was strictly enjoined till the symptoms of fever and irritation subsided.

---

unfavorable prognostic of the success attendant on lithotomy, yet is not an insuperable reason for its being altogether abandoned; for daily observation shews, that this organ, when indurated and enlarged, will frequently endure much violence with comparative impunity.

The symptoms characteristic of stony concretions in the bladder, and an indurated condition of the prostate gland, are sometimes confounded with each other. Hence will appear the necessity of discriminating betwixt these two diseases; and an attentive observer may generally form a tolerably accurate criterion, by attending to the nature of the discharge from the urethra; for, in a disease of the prostate gland alone, the discharge is generally a mixture of viscid mucus and urine, and, when connected with calculi in the bladder, it becomes frequently incorporated with sabulous matter.

A thickening of the coats of the bladder, even if it could be ascertained prior to the operation, yet ought by no means to prevent its being performed, unless connected with some other disease, for I have witnessed many cases of this description which terminated favorably.

"On

"On the 20th instant, three days after the operation, a degree of soreness and tension manifested itself in the lower part of the abdomen, which extended along the urethra, and which assumed the appearance of peritoneal inflammation. But, on a minute investigation, I was convinced, that the tension of the abdomen was caused by the parts of the wound connected with the operation being distended with inflammation, which wholly prevented the action of the bladder and voluntary power of the abdominal muscles from propelling the urine* through the aperture. Without hesitation, I passed a female catheter up the wound in perineo into the cavity of the bladder, and evacuated more than a quart of limpid urine of healthy appearance. This unusual mode of assisting nature in relieving herself, was found necessary to be repeated every eight or ten hours for several succeeding days, until the tension and inflammation of the parts connected with the wound had subsided; after which the urine returned through the artificial aperture with comparative freedom. About three weeks from the time of the operation, a little urine made its way, at intervals, by the channel of the urethra, and the man seemed gradually recovering; when on a sudden a new train of symptoms came on, accompanied with inflammation and swelling of the right testicle, attended with violent obtuse pain, which produced a slight degree of fever and constitutional irritation of the system. Ten leeches were applied to the inflamed scrotum, and cloths moistened in a solution of ammonia muriata in vinegar and water were kept constantly applied to the affected part; a brisk purgative draught was administered, and a scanty regimen enjoined; yet every precaution used to disperse the swelling and inflammation proved unavailing, and suppuration was announced by frequent rigors, and the structure of the testicle becoming less tense.

* I am induced to believe, that ischuria vesicalis, subsequent to the operation of lithotomy, is a very rare occurrence, and, so far as my inquiry extends, is scarcely noticed by any authors who have written on the after-treatment of patients laboring under this disease, and it being a circumstance which may be occasionally mistaken for peritonitis, seeing the symptoms, on a superficial inspection, bear some analogy to each other. Hence will appear the necessity, in every instance of abdominal affection, for the surgeon minutely to investigate the state of the bladder, and witness the discharge of urine from the wound or by the urethra. By strict attention to this particular, the morbid retention of urine may be distinguished from abdominal inflammation, and by the introduction of a female catheter through the wound into the bladder, the water may be discharged as often as required, and till the inflammation subsides, by which means the patient will be relieved; and by neglect of this mode of manual assistance, inflammation of the bladder and abdominal viscera would inevitably follow, for there is much reason for believing, that serious mistakes have sometimes ensued from this species of retention of urine.

A poultice was then applied, and renewed three times a-day, till a fluctuation of matter became perceptible, which was let out through an opening made with a lancet; the part soon healed, and the tension of the testicle gradually subsided; soon after which the left testicle became enlarged and painful, and assumed one smooth solid substance. Leeches and other topical applications were assiduously applied, as in the former affection, and a mixture, apparently of pus and urine, was regurgitated* by the urethra, which continued for the course of eight or ten days, and then the inflammation and swelling gradually disappeared. From this period the wound in perineo assumed a granulating and healthy appearance, and the urine was voided voluntarily through the urethra in increased quantity; and in the space of ten weeks, from the time of the operation, the wound was completely healed, and the man returned home in a state of apparent good health, being able to retain his urine in considerable quantity, and propel it at pleasure. On a minute examination of the state of the prostate gland at this period, by the finger in ano, its size appeared very much diminished from what it was prior to the operation.†

"I have lately had an opportunity of conversing with my patient, and he informs me, that some weeks past he parted with two small pieces of rough calculi by the urethra, and there is a deposit of sabulous matter in his urine, from which it appears probable that the disposition to the formation of stone still exists."

IV. *Case of Encysted Tumor of the Brain. By* A. J. Buchanan, M.D.—A girl, five years old, had the hooping-cough, the symptoms of which had been mild, but they

---

* I conceive it is not easy to explain the real cause of this malady of the testicles, nor do I find such an occurrence mentioned by writers on lithotomy: however, I will hazard a conjecture, that in all probability some irregular particles of calculi, separated from the urine, might become entangled behind the veru montanum, and obliterate the orifices of the seminal ducts, and in this way produce irritation of the contiguous parts, or by sympathy the vas deferens might participate therewith, and the inflammation be communicated to the testis; for it appears likely that the matter was regurgitated through this duct from the testicle to the urethra.

† I am inclined to believe, that, if the gorget had been used in this case, it would not have divided more than half of the left portion of the prostate gland, consequently the incision must have been quite inadequate for the extraction of the stone, and probably too small to admit the blades of the forceps without much violence; and, owing to the indurated structure of the gland opposing such a degree of resistance to its introduction into the bladder, it is more than probable that, during the attempt to accomplish this step of the operation, the prostate gland must have been severed from its connection with the membranous portion of the urethra, and the posterior part of the bladder become transfixed.

did did not perfectly subside. Under these circumstances Dr. Buchanan saw her. An emetic had not been taken during the previous course of the disease; an antimonial one was then ordered; though the paroxysms of the cough had become daily fewer, yet the health of the child had decreased.

" She spent her nights restlessly, did not take her meat as usual, and her stools were slimy and dark-colored. Her mother also assured me, that she had been much changed within these few days, in almost every respect—her toys afforded her no amusement—at one time she would become unusually lively, and, at another, insensible and torpid; and that, when free from either of these, she was peevish, fretful, and discontented. Such incongruous symptoms induced me to be more particular in my examination, and upon after-inquiry I learned, that, at all times since the accession of the hooping-cough, she appeared much disposed to sleep, but could enjoy none, latterly, from her incessant tossing and restlessness. This train of symptoms had become more urgent for two days past; the pulse was small, quick, and frequent, beating 135 in the minute; and Fahrenheit's thermometer, upon placing the bulb in the axilla, stood at 102°. Her tongue was white and furred, and her thirst considerable. An attendant told me, that, when more sensible in the night, she had complained of pain in the head. She was much reduced in flesh; her face was suffused with an unhealthy flush; there was much throbbing of the temporal artery, and the eyes were tender. These symptoms led me to think, that the child was laboring under an attack of the common infantile fever, and that all the appearances mentioned originated from, or were at least intimately dependent upon, the state of the alimentary canal. Cathartics being therefore indicated, I ordered a brisk one, which was immediately given. By next morning, the medicine had effected a very complete evacuation of the bowels, the greatest part of which was very fetid, and of a mucous appearance and consistence. She had vomited a quantity of light greenish fluid; the pulse was reduced to 100, without intermission; the heat was more moderate, and I began to form a favorable prognosis. My hopes were, however, delusive; for upon the succeeding morning a great change for the worse had taken place; the pulse still continued less frequent, and the febrile heat appeared recessant; but the pulsations were tremulous and irregular, and the extremities were cold and livid. The mouth soon became loaded with aphthæ, the respiration was stertorous, and she appeared to be comatose. These symptoms continued with exacerbation till the morning of the 14th, on which day, at eleven o'clock A.M., she died."

On examination *post mortem*, the cavities of the thorax and abdomen did not present any morbid changes that could account for the symptoms and death of the patient; but in the cavity of the cranium appearances of diseased alteration were found, to which the fatal termination of the case was attributed.

attributed. The state of the brain, its membranes, and vessels, are minutely described.

"On removing the skull-cap, the external surface of the dura mater appeared to be in a healthy condition; it was therefore (being divided on a level with the section of the skull all around, except at its posterior part,) thrown, along with the falx, in the usual way, backwards over the occiput. The other membranes being now exposed, several circular spots of a white milky color, and varying from about the size of a mustard-seed to that of a seven-shilling piece, were discovered scattered irregularly in several places over the surface of the brain. They lay chiefly in the furrows between the convolutions of the middle and the posterior lobes, although some smaller ones were found over the convexities of the convolutions, and a few of a middle size and bluish-white color on the anterior lobe of the right side. On the same side, and in the direction of the corda silvii, lay six of the largest kind, of a darker hue than the others. Upon raising the tunica arachnoides, which was done with difficulty, I clearly ascertained that these vesicles were situated between that membrane and the pia mater, and what appeared remarkable was, that they seemed to have little connexion with either, for I could easily raise them entire upon the end of the knife. They appeared to be surrounded with a thin membranous covering, and, when pressed between the finger and thumb, a quantity of fluid-like cream, but of a darker color, and more viscid consistence, intermixed with streaks of blood, was effused. Having ascertained every thing concerning these bodies that I thought worthy of notice, I now proceeded, in the usual manner, to dissect away the substance of the right hemisphere, on a level with the section of the cranium, in order to expose the centrum ovale minus. The brain cut very well for about one-third of the length of the hemisphere, but when I had reached to about the beginning of the middle lobe, an effusion of dark bloody fluid made its appearance. Carrying the division backwards, I brought into view a horizontal section of a circumscribed dark purple-colored circular body, of a homogeneous appearance, which, upon being washed, appeared well defined at its margin, inveloped in an inflection of the pia mater, and encircled by a thin layer of cineritious matter. It was, therefore, though apparently in the very centre of the medullary part, in fact exterior to the pia mater and cortical portion of the brain. Upon more particular examination, I observed, that this collection of morbid matter extended inwards, from the situation of the corda silvii, where I had observed the largest and darkest-colored of the small bodies upon the surface, and that it was fairly encysted; the sac being composed, as I have said before, of a prolonged duplicature of the pia mater; also, that that part of this membrane which surrounded it was opaque, thicker than natural, and painted in all directions with red vessels. This body in appearance was very similar to the spleen, although its substance was less compact, and its texture more delicate. Having examined the tumor in all directions,

 and and ascertained every thing I could possibly discover, I proceeded, with considerable expectation, to open the lateral ventricles, which, notwithstanding the previous dissection, still remained entire. They contained each a small quantity of aqueous fluid. The fornix was now divided at its anterior crura, and the velum interpositum, which exhibited a most beautiful appearance, exposed to view. The whole of it, but particularly the plexus choroides, was distended with vessels almost to bursting, and what are usually imagined to be hydatids, were clustered in great numbers round the latter part. I continued to dissect downwards, but could discover nothing further unnatural, except that the peduncles of the pineal gland were unusually large and prominent."

The only effects which, *à priori*, could be attributed to this extensive disorganization of the brain, was some degree of stertorous breathing and disposition to coma: the other symptoms were those of idiopathic fever. We do not forget that Dr. Clutterbuck has attributed all fever to a diseased state of the brain; and here is an instance of very extensive morbid change in that organ, the effect of which was idiopathic fever, an increased action of the arterial system, having been one of the chief characteristics of the disease. We do not mean to discountenance the examination of bodies after death, when we observe that less light has been thrown on the causes and symptoms of diseases by dissection than reasonably would be expected.

V. *Account of unusual Conformation of some Muscles and Vessels; by* ALEXANDER RAMSAY, M.D.—A transverse oblong muscle, stretching from the pectoral to the latissimus dorsi and teres major, has been seen by Dr. Ramsay; and he accounts for the liability of some subjects to swelling of the axillary glands on occasions of violent exertion, from the existence of this muscle. Three instances of unusual distribution of arteries have occurred within the observation of the author. A branch from the external iliac, exceeding the diameter of a crow-quill, and lying in the line where the trocar pierces in paracentesis of the abdomen, is described and represented in a small diagram. The wounding of this artery, which exists, according to the experience of Dr. R. once in 200 subjects, would be fatal, if the practitioner were not acquainted with the accident, and secured the vessel by ligature. But will this accident ever happen when the paracentesis is made on the *linèa alba?* The next deviation from the usual distribution of vessels is of an artery as large as a crow-quill, which traverses the middle of the trachea to be diffused on the right lobe of the thyroid. It is interposed where the trocar passes in tracheotomy. The third, is an instance of the anterior tibial artery, sent off higher than usual from the popliteal artery, and is subjected to the continual pressure of the popliteal muscle. It is a a question whether the pressure of the popliteal muscle on this changed position of the anterior tibial artery may excite an aneurismal affection.

VI. *On painful Subcutaneous Tubercle; by* WILLIAM WOOD, Surgeon.—The profession is indebted to Mr. Wood for bringing attention to an hitherto, we believe, unnoticed disease. He describes this disease to be a *Tubercle*, of a peculiar nature, in the subcutaneous cellular substance; met with in different parts of the body, but most frequently in the extremities. It is generally of the size and form of a flattened garden-pea; of a firm consistence, circumscribed, lying loosely in the cellular substance, immediately under the integuments, which retain their natural color and appearance. Generally it is only to be discovered by the touch; it is sometimes, however, so superficially seated as to form a visible prominence. Whether it is of slow or quick formation originally is not known; but, having acquired a certain size, it has no perceptible increase in many years. It is generally solitary, but in one instance three of these tubercles existed in the same patient. This tubercle becomes an object of chirurgical notice, from the acute pain to which it gives rise.

"So strongly is this pain represented by the patients, that we might be apt to imagine their statement exaggerated, did we not find them all concurring in the same representation, and most of them willing to submit to any operation that may be necessary to remove the cause of their pain. Two women in the lower rank of life came to me, even from a distance of above 30 miles, to have a consultation about this disease, and both of them expressed their willingness to submit to any temporary pain, however severe, which might remove their complaint.

"The pain to which this disease gives rise, is extremely acute in the tubercle, and extends from it to a considerable distance along the neighbouring parts; it is not constant, but occurs in paroxysms. In general, at the commencement of the paroxysm, the pain is slight, but gradually increases till it becomes excruciatingly severe, and it goes off in the same gradual manner, leaving the parts in the neighbourhood of the tubercle, for some time afterwards, sore to the touch, as if they had been bruised. The paroxysms vary in duration from ten minutes to upwards of two hours; but they seem to increase both in frequency and severity, in proportion to the length of time the disease has existed.

"Some of the patients have occasional intervals of ease for days or even weeks; in others the paroxysms occur several times in the course of one day. They generally come on spontaneously; but, in one of the cases which I met with, they were sometimes induced by by the friction of the clothes along the surface of the tubercle. They frequently attack the patient when asleep, in which case he is suddenly awoke by the severity of the pain.

"The degree of pain produced by touching the tubercle is different in different cases, all the patients whom I have examined allowing it to be freely handled, *in its ordinary state,* without feeling much uneasiness, but complaining of great aggravation of the pain, from either the tubercle or surrounding skin being even slightly touched *during the paroxysm.* Acute pain is produced at all times, by the tubercle being accidentally struck against any hard substance. One lady, of whose case I shall give a detailed account, conceives that the sensibility of the tubercle is materially affected by every change in the weather, whether from heat to cold, or from cold to heat. Several of the patients have stated, that they are sensible of an increase in the size of the tubercle *during the attack of pain,* and some of them have occasionally observed, at the same time, a degree of discoloration of the skin covering it, which became of a purplish or bluish cast; and, although I have had no opportunity of ascertaining the truth of these statements from my own observation, I have no reason to doubt their accuracy."

Extirpation of the *tubercle* is a radical cure; and, from its dimensions and situation, the operation must be easy and expeditious.

The slight attachment this insignificant tumor has with the surrounding parts, the paucity of blood-vessels and of nerves, renders the acute pain with which it is accompanied highly mysterious. Several cases are given, two of which we shall extract, as further illustrative of this singular disease.

*Case.*—" Miss ———, a lady of about 30 years of age, consulted my father, in the year 1809, on account of three tubercles, rather smaller than garden-peas, situated superficially under the integuments over the upper part of the gluteus maximus muscle. These were placed pretty near each other; they were quite moveable, and the skin covering them was of natural color and appearance. She complained of frequent attacks of extremely acute pain in these little tumors, which extended from them to a considerable distance along the neighbouring parts. These attacks sometimes occurred spontaneously, but were frequently also brought on by the friction of her clothes along the tubercles. She was recommended to try the topical application of mercurial ointment, but, this not being productive of any relief, she most anxiously desired the removal of the diseased parts. The three tubercles, along with a considerable portion of the integuments and of the cellular substance, were removed by my father, by two incisions. The wound healed by the first intention, and the lady has never since had any uneasiness or return of her disease."

*Case.*—" Mrs. Craig, about 28 years of age, came to me in March last, from a considerable distance in Fife, on account of a tubercle of the size and form of a flattened garden-pea, situated immediately under the skin over the middle of the right leg, on its outer side. It felt firm but moveable, and the skin retained its natural color and appearance. Above seven years previous to my seeing her, she had become subject to frequent attacks of pain in the right leg, and soon afterwards she accidentally discovered the little knot, which had undergone very little alteration in size from that time. For several years back she had been subject to several paroxysms of pain in the course of every 24 hours, varying in duration from ten minutes to nearly three quarters of an hour; these she said had become exquisitely severe; they frequently attacked her when asleep, in which case she suddenly awoke in a fright, and occasionally found that she was screaming out from the severity of the pain. The pain was not confined to the tubercle, but extended to some distance both up and down the leg. By her account, the tubercle frequently became *visibly increased in size during the paroxysm,* and the skin covering it, sometimes at the same time, assumed a purplish or bluish hue. The paroxysms had been gradually increasing both in frequency and severity. I removed the tubercle, without taking away any part of the integuments, and a very small portion only of cellular substance. At the first removal of the adhesive straps, the divided parts were found to have adhered, but there was a slight discharge of pus from the surface of the wound for ten or twelve days. She has not experienced the slightest uneasiness since the operation."

VII. *Are those Diseases attributed to Mercurial Action on the System of the Human Body, peculiarly and exclusively generated by it? by* John Chisholm, M.D. F.R.S.—The object of Dr. Chisholm is to prove that certain eruptive diseases, supposed to be the peculiar offspring of mercury, and treated of by the late Sir George Alley and Mr. Mathias under the denomination of hydrargyria, and mercurial disease, may arise from other irritants, as all the animal specific poisons, the poison of fish, and many mineral and vegetable substances, such as antimony, opium, camphor, &c. This proposition he endeavors to establish by arguments and facts. The elaborate, hypothetical, and perhaps desultory, train of reasoning on the action of mercury, the nature of scrofula, &c. we must forbear to go into, that we may be enabled to insert some valuable facts, which prove that the morbid appearances on the skin, believed to be excited by mercury, may arise from other sources.

The mercurial ulcer of Mathias has occurred to the negroes in the West Indies, without the agency of mercury. It is of a peculiar kind, is seen principally to affect negroes, and especially when lately imported from the coast of Guinea. Dr. Chisholm denies it to belong to the genus Herpes, and describes it in the following terms:

"T[illegible] ulcer generally makes its first appearance, either in the form form of a pimple, or a small vesicle, which, bursting, discharges a sanious matter or a limpid fluid, evidently possessed of a very irritating and corrosive quality, which shews itself by an intolerable itching, and a very speedy enlargement of morbid surface. Sometimes this ulcer originates from the irritation and pricking of external substances, which the negroes are exposed to in the course of their field-work, such as the lanugo of the sugar-cane, the spines of various wild plants, sharp stones, &c. But, I imagine, these can only be called exciting or occasional, although by the planters they are considered the principal remote, causes. Whatever the cause may be, the effect is the same, and always very surprising; for in the short space of eight-and-forty hours, an ulcerated surface will be formed of more than two inches in diameter. In the progress of this ulceration, some appearances take place which indicate an herpetic character; these are chiefly the corroding nature of the discharge, sometimes its color and consistence, the creeping manner in which the ulcer spreads itself, and its leaving distinct portions of the natural color apparently unhurt:—thus frequently, within the general compass of the ulceration, insulated portions of sound skin may be perceived, and which may continue so during the existence of the disease. From the very commencement of the disease, the portion of the limb near and around the ulcer becomes tumid, and, as the former advances, the latter enlarges; so that when the ulceration has gained a surface of six inches by three, that part of the leg will have increased fully three inches in its circumference. It is peculiar to this disease, that, if its duration should be long, the limb never loses the dimension it has acquired, the swelling consolidating and becoming permanent. The ulceration is seldom deep; but I have known instances of its penetrating to the bone, occasioning a caries, and at length such a morbid state of the limb as to render amputation necessary. It frequently happens, but from what cause I know not, that the whole, or by far the greatest part, of the ulcerated surface fills up, and cicatrizes in a surprisingly short time. This is, however, mere fallacy, for, in the course of one night, the whole of the surface, thus seemingly whole, will be corroded in the same manner as at first, and an ulcer larger than the former, and of a more unfavorable aspect, will be formed. In proportion to the repetition of these attacks, as well as the duration of each, is the enlargement of the limb; so that limbs of an enormous size, from this cause, are not unfrequently seen, which, except the ulceration in which they have originated, have all the appearance of the Barbadoes or glandular disease of Dr. Hendy. Now, as mercury could have no share in the production of it, this ulceration must have had a different source, although its nature may probably be the same as the mercurial, that is, a latent constitutional disease brought into action by an irritant. The affinity of this ulceration to Elcosis herpetica (Sauvages, tom. ii. 610) is evident indeed; but there are several discriminating features which manifest their want of identity. Thus, the surface of the ulcer before us is always, whether superficial or penetrating, of a bright red color, and always without any collection collection of matter of any description. Thus too, when the ulceration extends itself, no sloughing of morbid portions has been observed, notwithstanding the surprising manner in which the extension takes place; thus the sound and the ulcerated surface either imperceptibly unite, or the edges of the former look sharp and florid, and, as in the 'mercurial ulcer,' seem divided by a knife, and this, whether the ulceration extends or contracts, which it will do in a very astonishing and rapid manner. With the herpes serpigo, a very common, obstinate, and often distressing, disease within the tropics, and all the other species of herpes, it has no other affinity but its divergent and creeping quality; in every other respect it differs from them. In short there is an identity only between it and the mercurial ulcer; and yet we can account for this in no other way, that I am aware of, than by supposing, as I have already observed, a latent constitutional disease, which, in both cases, is brought to light by irritation."

This disease, so similar to the mercurial ulcer, appears to be excited by very trifling circumstances in peculiar idiosyncracies. The following case shews that a disease very similar to eczema mercuriale, was excited by opium.

"A young woman of about 26, of a remarkably sallow complexion, rather delicate form, and subject to a discharge of a very fetid nature from behind the ears, complained of excruciating pain in the right temple, unattended with any symptom of inflammatory affection, and which she herself attributed to cold. Observing nothing which could lead to any other conclusion than that she labored under some degree of rheumatism in the temporal muscle, I directed her to keep her head warm, and to take a powder composed of pulv. ipecac. comp. gr. v. and camphor gr. iij. twice or thrice in the day, with a mild aperient occasionally. The second day after she began the use of this medicine, her face swelled enormously, and a papular eruption appeared on its surface. The following day this extended to the neck, breast, back, and arms. The next day (fourth from taking the medicine) her whole body was swelled, red, and covered with the same eruption. The papulæ were lymphatic at first, but, soon drying, they became scabs, very offensive in color and odor; under the scabs or eschars fetid matter accumulated, which, throwing off the eschars, formed disagreeable and troublesome little ulcers. Fresh crops of papulæ daily took place, so that the distress of the patient became excessive; for, joined to the actual pain she suffered from inflammatory distension, the very offensive discharge from the surface, the want of sleep at night, the thirst, and other symptoms of febrile action (pulse generally 120), her mind became a prey to despondency. As in the former case, by means of purgatives and infusion of bark acidulated, with embrocations of the linimentum aquæ calcis, Ph. Ed which had a most soothing effect, she recovered. But there is a circumstance of interesting import in this case which must not be neglected. About the ninth day of her illness, the patient had taken, with a view to ease the the pain and promote sleep, a draught at night, in which were twenty-five drops of tinct. opii. The effect was precisely similar to the first attack brought on by the compound powder of ipecacuanha. She felt the whole surface of her body as it were on fire (fervida eruptio), a considerable fresh eruption took place, with swelling of the face, more especially, to such a degree as almost to close up her eyes. A purgative, on the following morning, gave her almost instant relief. It was not until the end of November, or nearly two months from the first attack, she was completely restored to health."

A third case is related by Dr. Chisholm, in which antimony was the excitant.

"A gentleman, about forty, was siezed with synochus, in a form by no means severe. After the usual evacuations, he took a powder composed partly of the pulvis antimonialis. In the course of the following night, his body became excessively red, a swelling ensued, and an eruption of a papulæ was soon after thrown out. Without paying much attention to these symptoms, I treated the disease as mild synochus, and he soon recovered. But a very general exfoliation of the cuticle took place, and for a length of time such a depression of mind, and such a degree of debility, were superinduced, as could not be accounted for as the effect of the original disease."

These facts are sufficient to shew that an inflammatory affection of the surface, similar to that which occurs during a course of mercury, may, in particular habits, be excited by very different means. Several such instances have fallen within our own observation. We recollect one that seems much in point, and might have been mistaken for mercurial erythema. A gentleman in the prime of life was so irritable with respect to the action of Copava Balsam, that a few doses of it invariably produced inflammation and papulous eruption very extensively on the skin. It will be seen that if this balsam had been given toward the decline of gonorrhœa, when it was the fashion to give mercury in gonorrhœa, that this patient would have been pronounced to have been suffering from mercurial erythema.

As Dr. Chisholm believes he has shewn that both the mercurial ulcer, and eruption, may arise from other causes, it is but justice to cite his concluding paragraph, containing suggestions on the nomenclature of these diseases.

"With respect to the ulceration, I can perceive no just reason for the exclusive appellation 'mercurial disease' to it, seeing there is cause for believing that mercury is not the only irritant capable of producing it. I feel rather disposed to employ the word *Elcosis* with the adjunct *Erithistica,* which indeed is almost the epithet given to it by Mr. Mathias, omitting the word mercurial, for it is an *ulceration from irritation.* Now, as to the eruption, the words

*Erythema*

*Erythema* and *Eczema*, singly, do not by any means seem expressive of the whole character of the eruption. It partakes of both, and cannot, with any appropriation of sense, be designated by one only. Eczema certainly, of the two words, gives the most significant idea of the disease, but it does so only as to the mode of eruption. The eruption is red, but so are many others; the eruption is wonderfully rapid, fervid, of a burning heat, and intolerable itchiness, but an eruptive disease may be so, and yet be destitute of a red color: an union of the two, therefore, seems to give the most compleie and definite idea of the disease, if the appellative common to the eruptive and ulcerous forms of the disease is added. Thus the ulceration may be denominated *Elcosis erithistica,* and its species or varieties designed by the name of the irritant peculiar to each; mercurialis, hyperoxygenosa, or intertropica, a veneno, &c. And thus too the eruption may be distinguished, *eczema, ereuthron, erethisticum;* and its species or varieties, mercuriale, antimoniale, ex opio, e camphora, a veneno, a frigore, &c. My view, in thus attempting to define these two diseases by a characteristic nomenclature, is most fully expressed by the very ingenious and learned critic on the 'Hydrargyria of Dr. Alley.' 'The appropriation of the term *eczema* to the profuse eruption of minute vesicles originating from irritants, and not the result of previous fever, according to Dr. Willan's method, seems to be convenient and advantageous. It is convenient, because it expresses to the learned something of the nature of the eruption; and it is advantageous, in a systematic view, because it comprehends several varieties of the same sort of eruption, resulting from different exciting causes.' "

(To be continued.)

